# Saliva Liquid Biopsy for Point-of-Care Applications

**DOI:** 10.3389/fpubh.2017.00077

**Published:** 2017-04-11

**Authors:** Katri Aro, Fang Wei, David T. Wong, Michael Tu

**Affiliations:** ^1^School of Dentistry, University of California Los Angeles, Los Angeles, CA, USA

**Keywords:** saliva, liquid biopsy, point-of-care, biomarker, cancer

## Abstract

Saliva is a non-invasive biofluid, which is easy to collect, transport, and store. Because of its accessibility and connection to systemic diseases, saliva is one of the best candidates for the advancement of point-of-care medicine, where individuals are able to easily monitor their health status by using portable convenient tools such as smartphones. There are a variety of scenarios with which saliva can be used: studies have been conducted on using saliva to measure stress hormones, enzyme levels, developmental disease biomarkers, and even cancer mutations. If validated biomarkers were combined with high-quality detection tools, saliva would open up a new frontier in high-quality healthcare, allowing physicians and patients to work together for real-time health monitoring and high-impact personalized preventative medicine. One of the most exciting emerging frontiers of saliva is liquid biopsy, which is a non-invasive means to assess the presence and characteristics of cancer in a patient. This article will review current basic knowledge of biomarkers, review their relation to different diseases and conditions, and explore liquid biopsy for point-of-care applications.

## Introduction

In the era of new diagnostic methods and treatment options, patient care is rapidly changing. There are many new paradigms in the evolution of modern healthcare: the White House has advocated for precision medicine, which tailors individualized treatment to the patient (1). Early detection is another emerging paradigm, which seeks to decrease patient morbidity and mortality by detecting disease at a phase where it is easily treatable. Early detection usually improves the success of treatment, prevents complications, and enhances patient prognosis. This is highlighted in common diseases affecting large populations such as cardiovascular diseases, diabetes mellitus, and various malignancies, as a recent review discusses (2). Precision medicine and early detection merge together with a third major paradigm: point-of-care diagnostics. Point-of-care diagnostics is a field of investigation that explores technologies that allows patients and health providers to gain actionable medical information rapidly and conveniently. Point-of-care diagnostics seeks to achieve “bed-side” diagnosis, removing the time delay that is caused by the conventional workflow of collecting samples and transporting them to a central lab for testing. The paradigm of point-of-care diagnostics joined to precision medicine and early detection paint a compelling vision of the future: one where doctors and patients can use small and portable devices to rapidly assess a patient’s health status, catching diseases extremely early and allowing ultracustomized treatment based on a patient’s personal characteristics.

One of the most critical questions that must be answered in point-of-care personalized medicine, however, is the question of which biomarkers to use for health monitoring. A biomarker is defined as a measurable, objective indicator of an individual’s normal and abnormal physiological state and indicates any change in that state. If these biomarkers are appropriately applied and patients are correctly instructed on how to respond to altered biomarker levels, this can be an immense benefit to rapid and personalized healthcare. Popular examples of well-selected and applied point-of-care biomarker tests is the detection of human chorionic gonadotropin in urine as a predictor of a woman’s pregnancy status, a test invented in 1968 and popularized through the off-the-shelf home pregnancy test kit (3). Another widely applied point-of-care biomarker test is the testing of glucose levels for diabetes monitoring through portable glucose meters. These success stories are extremely encouraging precedents for personalized point-of-care medicine, and physicians and scientists have hoped to widen the research of what we can detect at the point-of-care setting. Numerous studies have been conducted over the past decades on countless diseases, exploring clinically relevant biomarkers for the detection and follow-up of pathologies such as oral cancer (4), pancreatic cancer (5), lung cancer (6), ovarian cancer (7), and breast cancer (8).

**Figure 1:**
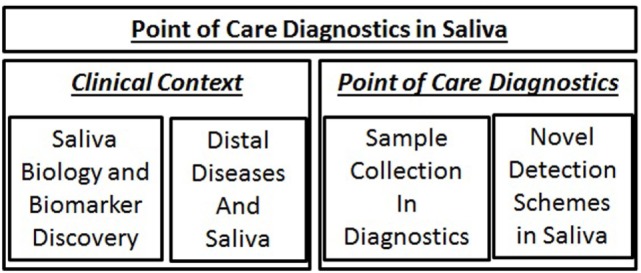
**Topics to be addressed in this article**. Discussion will begin with addressing the biology and clinical context of saliva diagnostics and then a brief survey of saliva collection and novel detection tools.

Since the dawn of the new millenium, research has paved the way for demonstrating that saliva is a highly viable biofluid for diagnostic application. Saliva includes various components, including DNA, RNA, proteins, metabolites, and microbiota. Saliva, as an inexhaustible biofluid, provides real-time data of the patient’s health status with a variety of possible translational applications. Saliva collection is straightforward, easily accessible and repeatable, and non-invasive. Moreover, the patient can provide the sample independently, even at one’s home without any extensive equipment and handling. In this targeted review, we present current knowledge and future aspects of utilizing saliva as a reliable biofluid for disease-specific biomarker development, describe some existing methods that have been advocated for being useful for the point-of-care context (Figure 1), with an extended exploration of the electric field-induced release and measurement (EFIRM) method.

## Salivary Biomarkers

In performing assessment of whether saliva biomarkers can be applied to clinical practice and used for diagnostic purposes, some key obstacles must be overcome. First, it must be credibly demonstrated that there are targets that are present in the salivary milieu that can be linked to a distal disease. Second, biomarker tests are not always perfectly accurate, and the scientific community is discussing how to best address the issue of false positives or false negatives. The only way to address this question is through a rigorous biomarker discovery process and a rigorous statistical validation of that biomarker. Third, a biomarker must not only be discovered and validated, but there must be also a credible mechanistic demonstration of constituents from a distal disease being transported to the oral cavity. While research is still being actively conducted to try to address these three key obstacles, much progress has been made.

Work by Li et al. (9) successfully discovered and analyzed the salivary transcriptome in 2004 in cell-free saliva, paving the way for further studies and explorations of extracellular nucleic acid targets. Hu et al. (9) also successfully performed proteomic profiling of the saliva. Further investigation over the past few years has also allowed scientists to elucidate the role of micro-ribonucleic acids (miRNAs) in saliva. miRNAs are single-stranded, non-coding RNAs, which in humans play a crucial role in cell differentiation, cell cycle progression, stress response, and apoptosis. miRNAs have a role in carcinogenesis and have been identified as potential biomarkers in different malignancies (10), including nasopharyngeal carcinoma (NPC) (11). A recent review outlines several aberrantly expressed miRNAs in NPC, which can be used as diagnostic and prognostic biomarkers and also as possible therapeutic targets (11). When the miRNA expression profile in NPC and furthermore in other malignancies has been fully understood, early diagnosis and even screening of the disease may become a reality. These earlier studies beginning in 2004 and recent studies help pave a way to saliva being a useful diagnostic medium for a variety of contexts:

1. *Systemic diseases*: many studies have been published to link salivary transcriptomic profiles to diseases affecting large populations: acute myocardial infarction (12–15), diabetes mellitus (16), Sjogren’s syndrome (17), developmental issues concerning premature neonates (18), cystic fibrosis (19, 20), and Parkinson’s disease (21–23). Based on a pilot study, matrix metalloproteinases may show elevated levels in saliva among patients with oral vesiculoerosive diseases acting as potential diagnostic markers or therapeutic targets (24). The use of saliva to detect cardiac troponin in patients showing suitable symptoms has been studied (12), but need further validation. In addition, salivary biomarkers have been studied among certain cancers, e.g., oral (4, 25), breast (8), pancreatic (5), ovarian (7), melanoma (26), gastric (27), and lung cancers (26).
2. *Oral hygiene and periodontal disease*: saliva also has an immediate effect on general hygiene and healthcare. Specifically, saliva has protective influence on dental structures and oral mucosa while containing several antibacterial components and buffering capacity. The composition of saliva varies according to a number of factors such as genetics, oral hygiene, general health, medication in use, tobacco use, alcohol consumption, and any previous treatment that might affect salivary gland function (e.g., surgery, radiation therapy). Cariogenic bacteria produce acids and lead eventually to caries (28). Periodontitis is a chronic inflammation of the periodontium. It is caused by persistent bacterial infection, which can lead to the loss of supportive material to the teeth, and it has currently high 47% prevalence in the adult population (29). The clinical diagnosis of periodontitis depends on time-consuming measurements, and the diagnosis is often delayed due to the lack of clear symptoms. As both caries and periodontitis can lead to tooth loss, the importance of preventive measures and early detection turn out as of high importance. Not only do these actions benefit individuals but also they are cost-effective. Ji and Choi (30) present in a recent review future insights to validate salivary biomarkers for the early diagnosis of periodontitis. Microbial DNA constitutes 30% of the salivary genome, leaving the rest 70% to be of human origin (31).
3. *Pharmacotherapy*: saliva can also be utilized in monitoring therapeutic drug level and treatment responses, for example, in persons with epilepsy (32). Furthermore, saliva may act as a monitoring tool, for example, to assess the levels of nicotine and cotinine in subjects who smoke (33, 34), to determine the possibility of drug abuse (35), and to excessive alcohol consumption (36).

These studies that have examined salivary constituents in disease coupled with mechanistic examination of the relationship of saliva with distal diseases (such as demonstrated by Lau et al. (37) for pancreatic cancer) show that obstacles to salivary testing for disease are steadily being eroded, and it may be only a matter of time before we have multiple thoroughly vetted biomarkers in saliva.

## An Emerging Frontier: Liquid Biopsy and Circulating Cells and DNA

In the quest to advance the state of the field in point-of-care cancer diagnostics, a recent push has been in the field of liquid biopsy, which seeks to provide a convenient method of diagnosing and identifying cancer types from biofluids, instead of conventional biopsy of tissue samples. The latter carries limitations in regards to tumor heterogeneity and also provides information only from the time of tumor sampling without the possibility for continuous sampling, which is more easily accomplished with liquid biopsies. Biofluid testing may also provide significant means for early detection when the disease shows no other clinical signs that would be visible or detectable. There are a few notable constituents that have been linked to cancer, which are targeted in the biofluid: the first notable constituent that has been targeted for liquid biopsy is the circulating tumor cells (CTCs) which are shed by the primary tumor into the bloodstream. The second notable constituent that has been targeted by liquid biopsy is circulating tumor DNA (ctDNA), which can be extracted or detected from a biofluid.

Many promising analyses of CTCs have developed, as outlined in a recent review (38). The utilization of a variety of techniques is diverse, ranging from mechanical techniques that can isolate CTCs based on the unique physical properties of cells to molecular techniques that isolate based on the surface markers of a tumor (39). However, concerns about their effectiveness exist due to the low concentrations of CTCs among blood cells, which means that either a large volume of sample may be required to be passed through an isolation system or an ultrasensitive method for detecting these cells be used. A further issue of CTC purification and testing for cancer diagnostics is the fact that CTCs possess heterogeneity, and as a result, the tumor cells isolated from a biofluid specimen may possess genetic alterations that do not accurately reflect the genetic profile of a primary tumor (38).

The second major constituent considered for liquid biopsy, ctDNA, is also being actively investigated with the hope that the obstacles typically present in CTCs would be minimized by targeting ctDNA instead. As opposed to the various purification techniques that require high sample volumes and specialized equipment, ctDNA targets can simply be isolated through traditional DNA extraction methods (40). On a clinical level, it should also be noted that ctDNA may also aid in other molecular diagnostics besides cancer, inasmuch as concentrations of circulating cell-free DNA can furthermore indicate several conditions besides cancer, such as autoimmune diseases, stroke, sepsis, trauma, and myocardial infarction (41, 42).

Both CTC and ctDNA targets for cancer profiling offer various advantages and disadvantages, and while there is no definitive answer regarding the future of liquid biopsy diagnostics, in general, it can be stated that ctDNA has had a greater ascendency in recent years since it is simpler to work with (inasmuch as it does not require the usage of specific extraction methods such as beads or microfluidics to achieve separation from a biofluid sample) (43). It should also be noted that much of the biology behind CTC and ctDNA is also under investigation for their precise relationship with distal cancers. For example, for ctDNA, it is hypothesized that they are stably found in biofluids because they are encapsulated in extracellular vesicles (EVs), which are membrane-bound structures and are released by cells into the circulation. Preliminary mechanistic studies, for example, have found that pancreatic cancer exosomes have been found to be transported to the oral cavity in a mouse model (37). If the links between distal cancers and the oral cavity can be thoroughly vetted and validated through investigation, it can help further our ability to precisely, effectively, and non-invasively diagnose cancers through bodily fluids.

## Saliva’s Relation to Virus-Associated Head and Neck Cancers

With the recent push for liquid biopsy and our constantly progressing understanding of salivary biomarkers, it seems logical that saliva liquid biopsy will be a logical next step. One such cancer that seems highly relevant for salivary testing is head and neck cancer. The estimated incidence of head and neck cancer is rising with nearly 690,000 new cases annually (44). Most of these are squamous cell carcinomas (SCCs), and 50% of the patients recur locally or at a distant site, which leads to a dismal median survival of less than a year (45). Most oropharyngeal cancer (OPC) cases in the Western World are caused by human papilloma virus (HPV), especially type 16 (46), and the incidence seems to be increasing (47) also among elderly (48). The prevalence of HPV infection in the healthy population is approximately 7%, of which 1% is attributed to HPV type 16 (49), without necessarily carrying an increased risk for developing a subsequent cancer. The reason why the prevalence among men is three to five times higher than in women (49, 50) needs further elucidation, but it is clear that HPV-induced OPC is male predominant (51). Individual’s capacity to eliminate their HPV infection without any further disease burden and furthermore a relatively low incidence of OPC have decreased the need and efforts to screen for this cancer. The US Centers for Disease Control and Prevention (52) presents still low HPV vaccination coverage figures in the US, although 14 million people are infected with HPV annually (52), carrying an increased risk for cervical cancer, anal cancer, and OPC. Despite the currently available HPV vaccine that targets also the oncogenic type 16, other HPV types and species have been advocated for developing OPC (53). Detecting HPV in saliva, which would serve as a biomarker, has gained much interest as its use in diagnostics and follow-up among these patients would be beneficial (54, 55), although the sensitivity in OPC needs improvement. Early detection of OPC would result in the possibility to single-modality treatment without exposing the patient to the toxicity and later side effects of oncological treatment. This aspect of treatment de-escalation among HPV-positive OPC is under research (56).

This emerging knowledge regarding liquid biopsy and biomarker targets may have radical implications for point-of-care saliva testing in our management of head and neck cancers. The Cancer Genome Atlas data report *TP53, PIK3CA, FAT1*, and *CDKN2A* as key mutations in head and neck SCCs (57). In concordance with these findings, Bettegowda et al. (58) reported that in plasma samples, approximately 70% of head and neck SCCs show detectable ctDNA mutant fragments, and Lebofsky et al. (59) concluded that the mutational profile of a tumor from a biopsy was consistent with ctDNA in 97% of cases. Furthermore, among these forms of cancers, Wang et al. (55) showed saliva to serve as a diagnostic medium for DNA detection. Epstein–Barr virus (EBV) has also been linked to NPC (60). NPC shows distinct geographical variation, as it is more common in Southeast Asia and Southern China. NPC is difficult to diagnose in its early stages due to the anatomical location, and while it carries high metastatic potential, it typically responds well to treatment. Still, despite treatment improvements over the years, worldwide NPC causes 50,000 deaths annually (61). A study showed 95% of NPC patients to show EBV-DNA in plasma correlating with the activity of the disease (62), a tendency also shown by others (63). It seems evident that surveillance of patient’s EBV levels may aid in the follow-up of patients with NPC. But the concern still remains for early-stage diagnostics.

## Point-of-Care Applications and Future of Salivary Diagnostics

As the basic knowledge regarding salivary biomarkers grows and the landscape of modern medicine advances, it is evident that salivary biomarkers will improve efficient clinical workflow. The capstone of any successful biomarker study is that the appropriate implantation of the biomarker test is a streamlined workflow for two important steps in a clinical workflow: sample collection and sample detection.

### Sample Collection: Methods of Collecting and Processing Saliva

While saliva is on the whole primarily water, there are still many constituents such as mucins, proteins, and enzymes that have the potential of interfering with test performance. Successful analysis of the salivary components requires an optimized process for sample collection, processing, and storage procedures. Partly due to diurnal variation, it is imperative that all these aspects are stable between various collection and analyzing points, especially when testing the same individual at different time points. Also, the consistency of saliva may alter within a person and between persons, which needs to be considered when detecting and validating possible new salivary biomarkers. If not correctly addressed, poor application of collection and sample processing can be particularly detrimental to the developing world, where resources for sample collection, diagnostic laboratory tests, and treatment are even more limited compared to an industrialized nation, and diagnostic tools with inadequate trained personnel may result in further misdiagnosis and malpractice. In a research performed in University of California, Los Angeles (UCLA), it has been established that the processing of collected saliva samples from patients should be performed within an hour of collection into a collection tube (64). After collection of the salivary sample from a patient, centrifugation is performed to separate cells from the saliva: the supernatant is clearly formed after centrifugation, separated from the pellet of cells from the whole saliva, and then stabilizing agents are added to these separated samples and are stored at −80°C for long-term preservation (64).

However, recent studies in salivary testing systems seem to show that saliva in the future may be more and more easy to collect, store, and transport. An example of this is in the RNA-Prosal, which consists of an absorbent that can be inserted into the mouth to collect saliva, and a filtered nozzle, which allows removal of cellular debris and salivary mucins that may disrupt biodetection (65). The study conducted in this work found that a streamlined collection and filtration unit was comparable to a conventional lab-based collection and centrifugation system. This is encouraging evidence that collection and processing issues that might be present in saliva diagnostic methods can be solved soon.

### Sample Detection: Techniques and Tools for Point-of-Care Salivary Biomarker Detection

Inasmuch as the field of biomarkers for saliva is vast, diverse, and everdeveloping, a wide stream of development that parallels investigations into useful biomarkers is the development of tools for point-of-care testing in saliva. One of the key requirements of this field for a tool is that it be appropriately designed to accommodate for the salivary matrix, which may have molecular constituents in a lower concentration than in other traditionally used biofluids. Various strategies have been taken to meet this requirement: some tools have molecule capturing and quantification techniques that allow them to have higher sensitivity than traditional tools, while some tools take steps to extract and enrich the amount of target molecules. This is an exciting field of salivary diagnostics that captures imagination. Many of these tools offer the promise of making personalized healthcare easily accessible to all patients. Some key technologies that are related to this are discussed below.

#### Photometric

Shetty et al. (66) presented in their study a portable biosensor to test salivary alpha-amylase, indicating sympathetic nervous system activity and showing individual’s stress level. The saliva collector is placed under the tongue to be saturated with saliva and then alpha-amylase reacts with a chemical with a change of color, which can be read with a portable optical readout device, all within 30 s. The complementary metal oxide semiconductor in smartphone cameras allows its detection of chemiluminescence (67). In addition, the vast processing and storing capacity in today’s smartphones could enable its use in self-made assays and monitoring, reporting results online. Zangheri et al. (67) developed a chemiluminescence imaging system utilizing a smartphone camera to quantify cortisol level in human saliva. The test requires a limited amount of saliva collected with a swab, and test results are available in 30 min. Carrio et al. (68) showed a drug-of-abuse detection test using smartphones, integrated with a lateral flow assay system. The possibilities for a smartphone in translational and point-of-care applications are tremendous, limiting the need for regular visits to health care providers and therefore also decreasing costs.

#### Electrochemical

Electrochemistry utilizes the specific electrical reactions or byproducts that result from a biological reaction and quantifies them to make an assessment of the biological state. Beginning with the advent of the Clark glucose electrode, which allowed the measurement of glucose levels by having blood glucose react with glucose oxidase, electrochemical techniques have been actively explored as a solution to aiding the management of diseases. This has been explored in the field of salivary diagnostics: Zhang et al. (69) presented a smartphone-based analysis of salivary alpha-amylase, providing quantitative results in 5 min. They showed positive correlation with the physiological state of the patients. These are compelling point-of-care tests with minimal effort regarding sample collection and processing, rapid readout, and data collection. Singh et al. (70) review exploratory studies of salivary cortisol levels, and Lee and Compton (71) also explored the usage of electrochemistry and novel carbon nanotubes for the detection of salivary biomarkers. These studies appear primarily to be tentative proof-of-concept examinations of salivary biomarkers and their potential for electrochemical, but they serve as examples in applying the field of electrochemistry to biodetection in saliva. The appeal of electrochemistry is that inasmuch it is mediated through only circuits and biochemical reactions; electrochemical devices can be easily miniaturized and still highly sensitive.

#### Electronic Nose

Using a wide variety of technologies such as metal oxide gas sensors or mass spectroscopy (72), electronic nose technology seeks to be able to capture volatile compounds in the air that may be related to a particular pathology. Fend et al. (73), for example, performed an initial investigation on the feasibility of detecting tuberculosis from the constituents of sputum samples that went into the air, comparing the results of electronic nose detection with traditional culture-based methods of assessment. Shih et al. (74) have also conducted studies on whether these electronic nose technologies can be used in a real-time detection scenario. While there seems to be a need for this branch of field to further advance in regards to practical oral point-of-care devices on relevant biomarkers, it seems that electronic nose technology has the potential to be used in portable investigations, as demonstrated by the work by Roine et al. (75).

#### Microfluidics

The field of microfluidics involves the microscale manipulation of fluids through small channels. This field of study is typically integrated with other biodetection methods, but it possesses high potential because it can be used for specialized manipulation of samples in a way to concentrate or extract the specific components of saliva that may be related to a disease. Examples of microfluidic technologies include the optoelectronic and microfluidic system developed by Zilberman and Sonkusale (76) for the detection of stomach cancer biomarkers, which allowed for unfiltered saliva to be analyzed for ammonium and carbon dioxide levels, which are correlated to the presence of *Helicobacter pylori* in the stomach. Another notable example that bodes well for the future of point-of-care diagnostics is the platform developed by Chen et al. (77) for the detection of bacterial pathogens, which allows saliva to be collected on a sample and then run through a miniaturized unit that can automate all the nucleic extraction, polymerase chain reaction (PCR) amplification, and detection steps for easy detection of pathogens.

All of these core technologies have been combined and used in creative fashions through multiple proof-of-concept studies, all with the goal of making biodetection as sensitive and efficient as possible. The various proofs of concepts displayed by the scientists and engineers who utilize these technologies show that a future where rapid detection of salivary biomarker targets at the point of care is possible.

## EFIRM: The Integration of Salivary Biomarkers and Efficient Biodetection

One of the most exciting recent proof-of-concept studies of point-of-care biomarker testing in saliva is work that has come out of research at UCLA. This work is a liquid biopsy technique called EFIRM. It is an electrochemical detection technique that utilizes a system of immobilized probes and readout enzymes to capture biomarker targets directly from saliva. Electric fields can facilitate nucleic acid hybridization (78, 79), and EFIRM is based on this principle (80), allowing selective hybridization of sequences to probes (81). After the active hybridization capture of a nucleic acid sequence in the biofluid is performed, a specific detector probe with a fluorescein label is hybridized to the remaining, unbound mutation sequence. At the last step, oxidation and reduction reactions are generated with a reporter enzyme and tetramethylbenzidine-based substrate solution. These reactions occur at the surface of the electrode, and the measure is used to quantify the detectable target sequence (82). If the ctDNA is captured in an EV and potentially difficult to access, EFIRM has also been shown to be able to be used for lysing the EV before the DNA inside it degrades (80). When performed, the EFIRM process can be performed rapidly in less than 30 min from a sample of saliva in a point-of-care fashion. Current versions of EFIRM require the use of power from wall outlets, but the method is simple enough to be used in a field setting with minimal equipment and battery power.

This EFIRM technology sets an exciting precedent for the future of point-of-care salivary diagnostics because of its high accuracy. A recent blinded study conducted by Wei et al. (83) explored the detection of epidermal growth factor receptor (*EGFR*) mutation in saliva among patients with non-small cell lung cancer with EFIRM technology. Targeting these EGFR mutations that are related to lung cancer, EFIRM was able to accomplish an astounding sensitivity and specificity of over 95% in a cohort of lung cancer patients, distinguishing the genetic makeup of cancers from a biofluid sample. The application of EFIRM shows many of the ideals that scientists and engineers have hoped for in a point-of-care saliva system: the biomarker selected was extremely correlated to a cancer state, the biodetection system was able to perform with high sensitivity and specificity, and detection was able to be rapidly (<30 min) performed with a minimal amount of sample (<200 μL of saliva or plasma sample). Aside currently used PCR-based technologies for oncogene mutation detection, EFIRM offers the possibility to detect biomarkers from biofluids more efficiently with less workload. Without the need for DNA extraction and nucleic acid sequence amplification, EFIRM can perform an analysis of the mutation status rapidly in less than an hour. This is vital when considering a reliable objective measure for point-of-care applications. Currently, in most cases, the mutational status in a patient is known only after analyzing a tissue specimen. To obtain that, one needs to perform invasive procedures. Also, when monitoring treatment response, and possible recurrences, time is the issue. Sequential saliva sample collection is far more rapid and cost-effective than, e.g., imaging. Also, current imaging modalities are not able to detect small tumor volumes or micrometastases preceding clinically evident lesions. As a proof of principle, a pilot study conducted in 17 patients with non-small cell lung cancer showed that saliva assays conducted using EFIRM perform accurately, non-invasively, and rapidly compared with biopsy tissue-based testing for *EGFR* mutation status and can be used in continuous monitoring during treatment (84). If the results of EGFR can be further extended, it will show that point-of-care salivary diagnostics are realities that we may see very soon.

These preliminary results presented by EFIRM are promising demonstrations. In order to further our understanding of diagnostics, it appears that the reasonable next step would be to run direct comparisons between EFIRM and other possible point-of-care techniques. For example, microfluidics possesses novel opportunities for purifying and isolating constituents from biofluids, and if microfluidic techniques for biodetection were compared with EFIRM on the same batch of samples, it would be a helpful study for assessing the relative merits of each point-of-care technology. Similarly, to assess EFIRM’s viability as a diagnostic tool in general, comparison studies could also be conducted of EFIRM with the gold standard methods of detecting ctDNA targets (e.g., droplet digital PCR or next-generation sequencing). These gold standard methods for detection are the techniques for detection that have been the most robustly tested on large clinical cohorts, but typically require the purification–extraction of DNA from large volumes of biofluid specimens. EFIRM’s feasibility and robustness as a method would be best confirmed by a definitive trial using both EFIRM and a gold standard method in parallel.

## Conclusion

This brief overview of the current knowledge of salivary biomarkers and their future role in point-of-care applications demonstrates the need for more advanced technologies. Since it is shown that liquid biopsy and saliva as a media for biomarker detection are suitable and desirable, the efforts to improve the sensitivity of detection are in demand. EFIRM-based liquid biopsy can bring high-sensitivity detection on a relevant biomarker with an extremely streamlined protocol, and as a result, point-of-care diagnostics seem like an exciting reality that is coming soon. There is much work to be done in all the facets of salivary research (i.e., biomarker discovery, biodetection methods), but the future seems closer than ever!

## Author Contributions

Substantial contributions to the conception or design of the work, drafting the work or revising it critically for important intellectual content, final approval of the version to be published, and agreement to be accountable for all aspects of the work in ensuring that questions related to the accuracy or integrity of any part of the work are appropriately investigated and resolved: KA, FW, DW, and MT.

## Conflict of Interest Statement

DW is a co-founder of RNAmeTRIX Inc., a molecular diagnostic company. He holds equity in RNAmeTRIX and serves as a company director and scientific advisor. The University of California also holds equity in RNAmeTRIX. Intellectual property that DW invented and which was patented by the University of California has been licensed to RNAmeTRIX.
